# Obesity, salivary glands and oral pathology

**DOI:** 10.25100/cm.v49i3.3919

**Published:** 2018-12-30

**Authors:** Ignacio Roa, Mariano del Sol

**Affiliations:** 1 Universidad de Talca Facultad de Ciencias de la Salud, Departamento de Ciencias Básicas Biomédicas. Talca, Chile.; 2 Universidad de La Frontera, Facultad de Medicina, Programa Doctorado en Ciencias Morfológicas. Temuco, Chile; 3 Universidad de La Frontera, Facultad de Medicina, Centro de Excelencia en Estudios Morfológicos y Quirúrgicos (CEMyQ). Temuco, Chile

**Keywords:** Saliva, dental caries, pathology, oral, obesity, overweight, xerostomia, salivary glands, periodontal diseases, microbiota, Saliva, caries dental, patología oral, obesidad, xerostomía, glándulas salivales, enfermedad periodontal, microbiota

## Abstract

Obesity has reached pandemic proportions in recent years. Not only adults suffer from the disease, but increasingly children and young people. One of the main causes of overweight and obesity is excessive food intake, in particular heavily processed carbohydrates. Obesity alters multiple organs, including the salivary glands, bringing functional alterations with it. Among researchers, the relation between obesity and tooth decay, periodontal disease and xerostomia is being debated. More and more scientific reports are drawing attention to the changes in the microflora of the oral cavity during obesity. All changes are closely related to the morphological and functional alterations of the salivary glands. This article review presents the current points of view regarding the impact of obesity on the health of the salivary glands, and how changes their functions influence other structures in the oral cavity.

## Resumen

La obesidad ha alcanzado proporciones pandémicas en los últimos años. No solo los adultos padecen la enfermedad, sino también cada vez más niños y jóvenes. Una de las principales causas del sobrepeso y la obesidad es la ingesta excesiva de alimentos, en particular los carbohidratos altamente procesados. La obesidad altera múltiples órganos, incluidas las glándulas salivales, y trae consigo alteraciones funcionales. Entre los investigadores, se está debatiendo la relación entre la obesidad y la caries dental, la enfermedad periodontal y la xerostomía. Cada vez más informes científicos están llamando la atención sobre los cambios en la microflora de la cavidad oral durante la obesidad. Todos los cambios están estrechamente relacionados con las alteraciones morfológicas y funcionales de las glándulas salivales. Esta revisión del artículo presenta los puntos de vista actuales sobre el impacto de la obesidad en la salud de las glándulas salivales, y cómo los cambios en sus funciones influyen en otras estructuras de la cavidad oral.

## Introduction

Obesity is a chronic disease recognized as a global epidemic in developed and developing countries ^1^ ^-^ ^3^. In the last decade, its prevalence has increased significantly, its comorbidities causing 4 million deaths worldwide in 2015 ^4^. Despite being a disease of multifactor etiology, the most common cause of obesity is excessive calories consumption ^5^. In the last 30 years, its incidence has more than doubled in children and quadrupled in adolescents ^6^, equally affecting both genders and different socioeconomic levels as well as all ethnic groups ^7^, jeopardizing health and life expectancy. A 20% increase in overweight suggests a 20% increase in the mortality risk ^8^. Together with its comorbidities, it is also recognized as the second main cause of death in the U.S., surpassed only by smoking ^9^ ^-^ ^11^. It is well known that changes in habits in the last 30 years, such as the wide availability of dense, highly palatable foods and more sedentary lifestyles, have driven the recent increase in obesity prevalence ^3^.

The increasing prevalence of obesity is a considerable threat to public health, due mainly to comorbidities such as type 2 diabetes, cardiovascular diseases and certain types of cancer.^12^ ^-^ ^15^ Diseases associated with metabolic disorders such as hyperlipidemia have become extremely common,^16^ which, together with insulin resistance, are associated with obesity ^17^. Other comorbidities that can occur as a result of obesity include sleep apnea, osteoarthritis, infertility, idiopathic intracranial hypertension, gastroesophageal reflux, among other pathologies, as well as a closer relation to a high incidence of dental caries, periodontal disease, xerostomia, among others. ^1^ ^,^ ^18^ ^,^ ^19^ Although this association is still under discussion, due to the dispariety of results presented by different authors ^1^.

**Figure 1:**
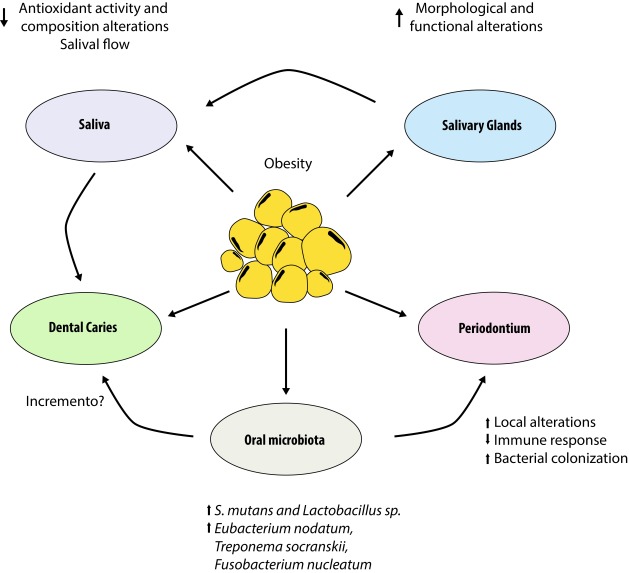
Interrelationship between obesity and oral cavity. It shows the relationship between obesity and alterations in the salivary glands and how they affect other components of the oral cavity, such as: production and salivary activity, tooth (dental caries), periondontium and microbiota.

One of the main structures affected by obesity and its comorbidities are the salivary glands, which are responsible for the secretion of a series of enzymes, growth factors needed for the biological balance of the oral cavity as well as for its protection. To date, the results observed differ in the possible effect of obesity on the morphology and function of the salivary glands, although its relation to several oral pathologies has been reported, such as tooth caries periodontitis and xerostomia, ^1^ ^,^ ^20^ ^-^ ^23^ intimately related to the action of saliva and glandular function (Fig. 1).

The aim of this article is to present an up-to-date review of the impact obesity has on the health of the salivary glands as well as on other tissues in the oral cavity that are closely related to salivary function.

## Obesity and salivary glands

### Salivary gland morphology

Obesity studies conducted on animal models have shown certain alterations in several organs, including the salivary glands. Inoue et al. ^(^ ^24^, reported a decrease in the weight of the salivary glands in rats with obesity induced by hypothalamic damage, results interpreted as an alteration that would cause a reduction in sympathetic nerve activity. In addition, it has been reported that genetically induced obesity (Zucker rats) promotes proinflammatory changes in the submandibular gland, and is also associated with rampant caries, despite there being no alterations in the architecture of the glandular tissue ^25^. Renzi *et al*. ^(^ ^26^, also reported changes in the submandibular gland, such as a reduction in glandular mass, hypotrophy of the adenomeres and an increase in the number of ducts after induction of hyperphagia by injury to the ventromedial nucleus of the hypothalamus. In obese subjects, a significant increase in the parotid gland has also been described, likely due to the storage of adipocytes in the parenchyma, whereas the submandibular gland does not seem to be affected ^27^.

Although studies relating obesity to salivary gland alterations are insufficient, other pathologies associated with obesity show alterations at the same level. Lilliu et al. ^(^ ^28^, related significant changes in the morphometric evaluation of the submandibular gland in diabetic patients, such as the widening of the size of the adenomas and granules, the reduction in mitochondrial size, the increase of the density of microfolds and protrusions throughout the luminal membranes, even though the function of the gland seems unaltered, suggesting that the morphological alterations reflect functional changes related mainly to secretory activity.

### Oxidative stress in salivary gland

It has recently been noted that an imbalance between the levels of reactive oxygen species (ROS) and antioxidants can play a key role in the development of pathologies in the salivary glands ^29^ ^,^ ^30^. It has been described that the parotid and submandibular glands in rats react differently when exposed to insulin resistance induced by a fatty diet, with the parotid gland being the most affected ^31^. On the other hand, Pannunzio *et al*. ^(^ ^32^, studying children with overweight and obesity, showed alterations in the concentrations of phosphate, free sialic acid and proteins, as well as in the peroxidase activity, conditions favorable to the development of dental caries.

In models of obesity induced in animals by monosodium glutamate (MSG), a significant increase in the content of substances reactive to thiobarbituric acid and a significant reduction in the activity of superoxide dismutase and catalase have been observed, which suggest an imbalance in the pro-oxidant and antioxidant systems and the onset of oxidative stress, ^16^ ^,^ ^33^, which may indicate an imbalance in the pro-oxidant and antioxidant systems and the onset of oxidative stress. Alterations observed in other organs such as kidney and liver ^34^.

Additionally, oxidative stress has been linked to obesity and cancer, which combined with angiogenesis, chronic inflammation, and the interaction of proinflammatory cytokines, as well as hormones and adipokines (leptin, insulin, adiponectin, growth factors, estrogen and progesterone) may be involved in altering the cellular metabolism, contributing to tumor development and progression ^35^.

### Obesity and saliva

Modéer *et al*. ^(^ ^36^, indicated that childhood obesity is associated with the reduction of the flow rate of stimulated whole saliva compared to individuals of normal weight (1.2 *vs.* 2.0 mL/min, *p* <0.001), which is linked to dental caries, reinforcing even more the negative effect of obesity on oral health. Based on the above, the proinflammatory cytokines derived from adipocytes and macrophages that have accumulated in the adipose tissue can negatively affect the function of the salivary glands due to low-grade chronic inflammation in the gland ^37^. Moreover, increased levels of proinflammatory cytokines have been reported in the crevicular fluid in obese adolescents in comparison with subjects of normal weight ^38^ ^,^ ^39^, with a hyper inflammatory reaction being observed in the periodontal tissue, similar to what was described in obese adults by Flink *et al.* ^(^ ^22^, which suggests that inflammatory mediators have an important role in the hypofunction of the salivary glands in obese individuals. 

According to the characteristics of the saliva, obese people present changes in the concentration of sialic acid, phosphorus and peroxidase activity, as well as a reduced flow of stimulated saliva, which is closely related to tooth caries and periodontal disease, so there is sufficient evidence to state that the saliva of obese and non-obese subjects is different ^40^. On the other hand, salivary changes, such as in concentrations of phosphate, sialic acid, proteins and immunoglobulins and peroxidase activity, could explain the greater likelihood that obese children are at increased risk of dental caries. ^41^

Analyzing total concentrations of different salivary components were found that the concentrations of total protein, amylase, urea, phosphate, triglycerides and calcium were similar between stimulated and non-stimulated saliva in children of normal weight, overweight and obese. However, the concentrations of urea, phosphate and calcium differed significantly between the stimulated and non-stimulated saliva in the normal weight and obese groups, with the lowest values for stimulated saliva ^42^ (Table 1).

**Table 1:** Relevant alterations caused by obesity in salivary glands. It shows the main morphological and functional changes in animal and human model.

Reference, Study	Results	Salivary gland	Subject
Inoue *et al*. ^(^ ^24^ ^)^	Weight glands decrease	-	Experimental model (rats)
Mazaffari *et al*. ^(^ ^25^ ^)^	Proinflammatory changes	submandibular	Experimental model (rats)
Renzi *et al*. ^(^ ^26^ ^)^	Glandular mass decrease, Acinus hypertrophy, ducts increased	submandibular	Experimental model (rats)
Bozzato et al. ^(^ ^27^ ^)^	Adipocytes increased	parotid	-
Pannuzio *et al*. ^(^ ^32^ ^)^	Phosphate, sialic acid, protein, concentration alterations peroxidase activity	-	Human (childrens)
Hordiienko *et al*. ^(^ ^33^ ^)^	Increase in thiobarbituric acid reactive substances	-	Experimental model
Beregova *et al*. ^(^ ^16^ ^)^	Increase in thiobarbituric acid reactive substances		Experimental model
Modéer *et al*. ^(^ ^36^ ^)^	Decrease total stimulated salivary flow rate	-	Human (childrens)
Choromanska *et al*. ^(^ ^40^ ^)^	Total stimulated salivary flow rate. Phosphate, sialic acid and peroxidase activity decrease	-	Human
Guaré *et al*. ^(^ ^41^ ^)^	Phosphate, sialic acid, protein concentration, Ig and peroxidase activity decrease	-	Human (childrens and adolescents)
de Campos *et al*. ^(^ ^42^ ^)^	Urea, Phosphate, calcium concentration and stimulated salivary decrease	-	Human (childrens)

## Obesity and prevalent pathologies of oral cavity

Salivary glands and saliva plays a significant role in maintaining oral health, helping to build and maintain the health of soft and hard tissues. When saliva flow is reduced, oral health problems such as dental caries and oral infections can develop.

### Obesity and dental caries

In healthy teeth, the loss of minerals is balanced with the mechanisms of saliva repair, so if saliva is present in less quantity and quality, we can contribute to a carious process. While the link between dental caries and obesity is not clear, the literature indicates that obesity is certainly associated with the appearance of early dental caries in childhood and puberty ^43^ ^-^ ^46^. Although these two pathologies coexist over time and share common etiological factors, this would partly explain the lack of association found by some authors when studying diverse populations ^47^ ^-^ ^52^. Modéer *et al*. ^36^,reported that obesity in children/adolescents was correlated significantly with the number of tooth surfaces affected by caries, as well as plaque and gingivitis indices. Studies conducted on adults with severe overweight, obesity and coexistent diabetes indicate a significantly higher frequency of caries than in the control group of people with diabetes who are not obese ^53^ ^,^ ^54^. Data are similar to those reported by Yao et al. ^55^, who described a significant effect of obesity on the prevalence of dental caries in primary schoolchildren in China. However, it is difficult to determine clearly whether this is due to overweight or diet and hygiene issues. According to Prpić & Pezelj-Ribarić ^56^, the result of an unbalanced carbohydrate-rich diet that stimulates the development of *Lactobacillus* sp. and *Streptococcus* is that it promotes caries in humans. On the other hand, studies conducted by Costa et al. ^57^, confirm a correlation between obesity and dental caries; the study by these researchers on a group of children (average age 6 years) from low-income families, revealed that more than 50% of the participants had caries, and 25% of these children were obese. The socioeconomic level of the family, however, was the strongest factor in determining the existence of dental caries.

Lehmann-Kalata *et al*. ^(^ ^58^, reported a higher incidence of dental caries, and a worse state of the gums and oral hygiene in obese patients than in those of normal weight. Additionally, the latter had a significantly greater amount of stimulated and non-stimulated saliva than the obese patients, there being a statistically significant correlation between the increase in *Streptococcus mutans* and *Lactobacillus* spp. levels in obese patients. Farsi et al*.* indicated different values, establishing that for the primary and permanent teeth combined, the children with a higher body mass index (BMI) and waist circumference presented a lower prevalence of caries (*p* <0.05) ^59^. For their part, Cereceda et al*.* ^(^ ^52^ *,* found no association between dental caries and obesity/overweight; these researchers studied a group of students from 5 to 15 years of age in Chilean public schools at the middle socioeconomic level, finding no link between BMI and tooth decay, or between genders.

In their systematic review, González *et al.* ^(^ ^45^ *,* showed that the link between dental caries and obesity/overweight can be explained by the recorded increase in weight being due to diet, mainly the high frequency of sugar consumption and snacks between meals, which increases the number of cariogenic microorganisms. The lack of association between these two variables, however, may be due to problems in the sample size, related to a short follow-up period, and underestimated values that do not report injuries or tooth loss due to caries. 

On the other hand, the inverse relation between caries and obesity/overweight ^60^ could be because as the decay progresses and becomes more painful, it limits the masticatory function, thereby reducing food intake, in addition to biological, genetic, socioeconomic, cultural, dietary and environmental factors ^49^ ^,^ ^61^ ^)^ .

Salivary function is reduced in patients with obesity and overweight, which could affect the course of dental caries. There are many studies carried out in which a relationship between obesity and dental caries is established, other authors indicate that being pathologies that coexist over time and share etiological factors, this relationship could not be established, due to the multifactorial nature of dental caries. The decrease in flow and salivary quality in obese patients is clear, which undoubtedly will cause some effect in the process of remineralization, an important factor in the progression of dental caries. The need for more studies, both experimental and prospective, could help explain this association.

### Obesity, oral microbiota and periodontium

Healthy human microbiota is largely composed of microorganisms ^62^, and there are different factors that influence its composition, such as age, diet, antibiotics and most of the elements of a modern lifestyle, as well as certain diseases. From birth, the microbiome and the host’s immune system co-develop and are mutually interdependent ^63^. Thus, the microbiota shapes the development of the immune system and in turn the immune system sets the composition of the microbiota, as observed between sustained changes in the intestinal microbiota and its link to obesity ^64^ and insulin resistance ^62^. Other studies have described an altered microbiological colonization in the intestine of obese subjects, indicating that these have more Firmicutes and relatively fewer Bacteroidetes in the intestine than participants of normal weight ^65^ ^,^ ^66^.

To date there has been no clarity in the relationship between obesity and oral microbiota. However, in studies on animal models, it has been reported that obesity interferes with the immune system’s ability to respond appropriately to infection by the periodontal pathogen *Porphyromonas gingivalis* ^67^. In addition, an improved colonization of *Tannerella forsythia* in subgingival biofilm has been described in obese subjects ^68^. The circulating adipokines can influence the immune response at mucosal level in both the oral cavity and in the intestine, thereby affecting microbial colonization. For their part, Ley *et al*. ^(^ ^63^, reported that obese individuals present differences in their gastrointestinal microflora compared to those of normal weight, inferring in addition that the flora changes when weight is lost. Other authors ^68^ ^-^ ^70^ have indicated that the oral cavity in obese individuals has higher levels of several bacteria than in non-obese controls, and it seems likely that those bacterial species could serve as biological indicators of an overweight condition developing. Shillitoe *et al* ^(^ ^71^ *.* point out that there is variation in bacteria of the oral cavity in obese patients after bariatric surgery. The existing alterations in the microbiota of obese individuals may be related to the concentration of circulating adipokines, which can influence both the immune response in the level of mucosa in the oral cavity and in the intestine, thereby affecting microbial colonization ^70^. 

With respect to periodontal bacteria, Maciel *et al.* ^(^ ^72^, established that obese patients with chronic periodontitis had higher levels and/or greater proportions of several periodontal pathogens than those of normal weight, including *Aggregatibacter actinomycetemcomitans*, *Eubacterium nodatum, Fusobacterium nucleatum ss vincentii, Parvimonas micra, Prevotella intermedia, Tannerella forthytia, Prevotella melaninogenica* and *Treponema socranskii*. The proportions of most of these pathogens, as well as *Campylobacter rectus* and *Eikenella corrodens*, increased at the disease sites of obese patients compared to those of normal weight. Lehmann-Kalata et al. ^(^ ^58^ , have reported a statistically significant correlation between increased levels of *Streptococcus mutans* and *Lactobacillus* spp. in obese patients compared to patients of normal weight.

There is sufficient evidence to the association between obesity and periodontal disease is establish a significant positive association between the two ^73^; Modéer et al. ^36^, reported childhood obesity as being associated with increased gingival inflammation compared to children of normal weight (*p* <0.001). Possible causes of how obesity affects the periodontal tissues may be due to the secretion of proinflammatory cytokines from the fatty tissue ^74^ ^-^ ^76^; in addition, the expansion of this tissue during weight increase would restrict the blood vessels, causing the migration of macrophages towards the periodontium. The combination of the previously mentioned situations may induce a low-grade generalized chronic inflammation, possibly accompanied by hypertension, exacerbating the obesity- induced inflammatory load ^77^. Using the BMI as a parameter, Saito *et al*. ^(^ ^78^, in a study conducted in Japan, demonstrated a statistically significant correlation between BMI and the depth of the periodontal pockets in women with obesity, and showed that a BMI over 30 kg/m^2^ increased the risk of periodontitis more than four times. Other Japanese studies that analyzed the periodontal state using the Community Periodontal Index of Treatment Needs (CPITN), evaluating the needs for periodontal treatment, demonstrated a positive correlation between the exacerbation of the symptoms that indicate a significant progression of periodontal disease and the increase in body weight measured by the BMI. Although in experimental studies of periodontitis induced in animals, it was noted that there were no significant differences in the loss of alveolar process bone between obese and normal animals^79^.

For their part, Zuza *et al*. ^80^, studying children between 5 and 10 years of age, described the obese children as showing significant proportions of degrees one and three on the Community Periodontal Index (CPI) (44.2% and 7.4%, respectively) compared to subjects of normal weight (*p* <0.05). The Visible Plaque Index (VPI) was similar between the two groups (*p* >0.05). Bleeding on probing was greater in obese patients than in children with normal weight (*p* <0.05), which may indicate that obese children are more predisposed to periodontal disease. Similar results were found by Scorzetti et al. ^(^ ^81^, who found an link between obesity and the indicators of periodontal risk in children, such as plaque deposits and bleeding on probing. With respect to the association between periodontitis and obesity in adolescents, Cavalcanti *et al*. ^82^, after analyzing 559 individuals, established that 18.6% were overweight and 98.4% had some form of periodontal change such as bleeding (34.3%), calculus (38.8%), shallow pocket (22.9%) and deep pocket (2.3%), and that there was a relation between the presence of periodontal changes and obesity (*p* <0.05).

Obesity and its important inflammatory component undoubtedly contribute to increasing the severity and sequelae of periodontal disease. As previously described, there are many studies that describe the positive association between overweight/obesity and periodontal disease, mainly due to the constant action of chemical mediators secreted from the fatty tissue in the oral cavity, which creates a general pro-inflammatory environment and contributes to an increase in periodontal disease. This is added to the decreased salivary secretion, which plays an important anti-bacterial and anti-inflammatory role locally.

## Conclusion

This review describes the main underlying mechanisms around the influence of obesity on the morphology and function of the salivary glands, and how these alterations impair the functioning of other components of the oral cavity, being associated with pathologies of high prevalence such as caries and periodontal disease. Given the increase in the prevalence of the population with overweight/obesity all over the world, these are important aspects to consider.

The background presented should induce cooperation between physicians and dentists to increase awareness about health and improve the conditions of the oral cavity in patients with obesity and overweight.

More research is needed in this area, given how few morphological, ultrastructural and functional studies there are regarding the obesity-related changes that occur in the structures of the oral cavity, particularly in the salivary glands. These morphofunctional studies together with studies focusing on other aspects would make it possible to design new pharmacological therapies that would help mitigate the effects caused by obesity on the oral cavity. 
