# Supplementary material for: Obesity, salivary glands and oral pathology
Source: Colomb Med (Cali). 2018 Dec 30;49(4):280–7. doi: 10.25100/cm.v49i3.3919 (PMC6342082; doi:10.25100/cm.v49i3.3919)
Supplement: Related texts

# Review article: Obesidad, glándulas salivales y patología oral

## Introduccion

La obesidad es una enfermedad crónica reconocida como una epidemia global extendida tanto en países desarrollados como en vías de desarrollo ^1^^-^^3^. En la última década, su prevalencia ha aumentado significativamente, causando 3.4 millones de muertes a nivel mundial en el 2010 ^4^. La obesidad a pesar de ser una enfermedad de etiología multifactorial, presenta como causa más común el consumo excesivo de calorías ^5^. En los últimos 30 años, su incidencia ha aumentado a más del doble en niños y se ha cuadriplicado en adolescentes ^6^, afectando a ambos sexos y distintos niveles socioeconómicos así como a todos los grupos étnicos por igual ^7^; comprometiendo la salud y la expectativa de vida. Un 20% de aumento de sobrepeso sugiere un 20% de aumento en la tasa de mortalidad ^8^. Es así como ha sido reconocida como la segunda causa principal de muerte prevenible, superada sólo por el tabaquismo ^9^^-^^11^. Es bien sabido que los cambios en los hábitos en los últimos 30 años, como la amplia disponibilidad de alimentos densos, altamente palatables y estilos de vida más sedentarios han impulsado el reciente aumento de la prevalencia de la obesidad ^3^. 

La creciente prevalencia de la obesidad representa una amenaza importante para la salud pública, debido principalmente a comorbilidades tales como la diabetes tipo 2, las enfermedades cardiovasculares y ciertos tipos de cáncer ^12^^-^^15^. Enfermedades asociadas a desórdenes metabólicos tales como la hiperlipidemia, se han vuelto extremadamente comunes ^16^, las cuales, en conjunto a la insulino resistencia, se encuentran asociadas a la obesidad ^17^. Otras comorbilidades que podrían ocurrir como resultado de la obesidad incluyen apnea del sueño, osteoartritis, infertilidad, hipertensión intracraneal idiopática, reflujo gastroesofágico, entre otras patologías, así como una mayor relación con una alta incidencia de caries, enfermedad periodontal, xerostomía, entre otras ^1^^,^^18^^,^^19^. Aunque esta asociación aún está en discusión, debido a la disparidad de resultados presentados por diferentes autores ^1^.

Una de las principales estructuras afectadas por la obesidad y sus comorbilidades son las glándulas salivales, quienes son las responsables de la secreción de una serie de enzimas, factores de crecimiento necesarios para el equilibrio biológico de la cavidad oral, así como para la protección de ésta. Hasta ahora los resultados observados difieren en el posible efecto de la obesidad ante la morfología y función de las glándulas salivales, aunque ha sido reportada su relación con diversas patologías orales, tales como caries, periodontitis y xerostomía ^1^^,^^20^^-^^23^, íntimamente relacionados con la acción de saliva y la función glandular (Fig.1).

**Figura 1:** Interrelación entre obesidad y cavidad bucal. Muestra la relación entre la obesidad y las alteraciones en las glándulas salivales y cómo afectan a otros componentes de la cavidad oral, tales como: producción y actividad salival, diente (caries dental), periondonto y microbiota.

El objetivo de este artículo es presentar una revisión actualizada del impacto que tiene la obesidad en la salud de las glándulas salivales, así como en otros tejidos de la cavidad oral que están estrechamente relacionados con la función salival.

## Obesidad y glándulas salivales

### Morfología de las glándulas salivares

Estudios de obesidad realizados en modelos animales, han evidenciado ciertas alteraciones en diversos órganos, entre ellos las glándulas salivales. Inoue *et al.* reportaron una disminución en el peso de las glándulas salivales en ratas con obesidad inducida por daño hipotalámico, resultados interpretados como una alteración que induciría disminución en la actividad de los nervios simpáticos ^24^. Además, se ha reportado que la obesidad inducida genéticamente (ratas Zucker), promueve cambios proinflamatorios dentro de la glándula submandibular, además de estar asociado a caries rampantes, a pesar de esto no vieron alteraciones en la arquitectura tisular ^25^. Por otra parte, Renzi *et al*. ^26^ reportan cambios en la glándula submandibular, tales como disminución de la masa glandular, hipotrofia de los adenómeros, y aumento del número de conductos, luego de la inducción de hiperfagia por lesión del núcleo ventromedial del hipotálamo. En sujetos obesos también ha sido reportado un aumento significativo de la glándula parótida probablemente por un almacenamiento de adipocitos en el parénquima, mientras que la glándula submandibular parece no ser afectada ^27^.

Si bien no hay suficientes estudios que relacionen obesidad con alteraciones de las glándulas salivales, otras patologías asociadas a obesidad, muestran alteraciones a nivel de las mismas. Lilliu *et al.*^28^, relatan cambios significativos en la evaluación morfométrica de glándula submandibular de pacientes diabéticos, tales como ampliación del tamaño de los adenómeros y gránulos, la reducción del tamaño mitocondrial, el aumento de la densidad de micropliegues y protrusiones a lo largo de las membranas luminales, aún cuando la función de la glándula parece inalterada, sugiriendo que las alteraciones morfológicas reflejan cambios funcionales principalmente en relación con la actividad secretora.

### Estrés Oxidativo en glándulas salivares

 Recientemente se ha visto que un desequilibrio entre los niveles de especies reactivas de oxígeno (ROS) y antioxidantes pueden jugar un rol clave en el desarrollo de patologías en las glándulas salivales ^29^^,^^30^. Se ha descrito que la glándulas parótida y submandibular de ratas reaccionan de manera diferente cuando se exponen a una condición de resistencia a la insulina inducida por dieta rica en grasas, siendo la glándula parótida la más afectada ^31^. Por otra parte Pannunzio *et al*. ^32^, al estudiar niños con sobrepeso y obesidad, mostraron alteraciones en las concentraciones de fosfato, ácido siálico libre y proteínas, y en la actividad peroxidasa, condiciones favorables para el desarrollo de caries. 

En modelos de obesidad inducida por glutamato monosódico (GMS) se ha observado un aumento significativo del contenido de sustancias reactivas al ácido tiobarbitúrico y una disminución significativa en la actividad de la superóxido dismutasa y la catalasa, lo cual indica el desequilibrio de los sistemas prooxidantes y antioxidantes y el desarrollo de estrés oxidativo ^16^^,^^33^, lo cual indicaría un desequilibrio de los sistemas prooxidantes y antioxidantes y el desarrollo del estrés oxidativo. Alteraciones observadas en otros órganos tales como riñón e hígado ^34^. 

Adicionalmente, el estrés oxidativo se encuentra relacionando obesidad y cáncer, las que junto a la angiogénesis, inflamación crónica, y la interacción de citocinas proinflamatorias, además de hormonas y adipoquinas (leptina, insulina, adiponectina, factores de crecimiento, estrógeno y progesterona) estarían implicados en la alteración en el metabolismo celular, participando en el desarrollo y progresión tumoral ^35^.

### Obesidad y saliva

Con respecto al flujo salival, Modéer *et al*. ^36^, indicaron que la obesidad infantil se asocia con la reducción de la tasa de flujo de la saliva total estimulada cuando son comparados con individuos normopeso (1.2 *vs* 2.0 mL/min, *p* <0.001), asociándose con caries, fortaleciéndose aún más el efecto negativo de la obesidad sobre la salud oral. En base a esto, las citoquinas proinflamatorias derivadas de adipocitos y macrófagos que se han acumulado en el tejido adiposo pueden afectar negativamente a la función de las glándulas salivales debido a la inflamación crónica de bajo grado en la glándula ^37^. Por otra parte, han sido informados niveles aumentados de citocinas proinflamatorias en el líquido crevicular en adolescentes obesos, en comparación con sujetos de peso normal ^38^^,^^39^ observándose una reacción hiperinflamatoria en el tejido periodontal, similar a lo descrito en adultos obesos por Flink *et al.*^22^, lo que sugiere que los mediadores inflamatorios tienen un rol importante en la hipofunción de las glándulas salivales en los individuos obesos.

Con respecto a las características de la saliva, las personas obesas presentan cambios en la concentración de ácido siálico, fósforo y actividad de la peroxidasa, así como un menor flujo de saliva estimulada, esto se encuentra íntimamente relacionado con caries y enfermedad periodontal, indicando que hay suficiente evidencia para afirmar que la saliva de sujetos obesos y no-obesos es diferente ^40^. Por otra parte, los cambios salivales, como las concentraciones de fosfato, ácido siálico, proteínas e inmunoglobulinas y la actividad de la peroxidasa, podrían explicar la mayor probabilidad de que los niños obesos presenten mayor riesgo de caries dental ^41^. 

Al analizar concentraciones totales de distintos componentes salivales, se encontró que las concentraciones de proteína total, amilasa, urea, fosfato, triglicéridos y calcio, fueron similares entre saliva estimulada y no estimulada en niños con peso normal, sobrepeso y obesos. Sin embargo, las concentraciones de urea, fosfato y calcio difirieron significativamente entre la saliva estimulada y no estimulada, de los grupos de peso normal y obesos, con los valores más bajos para la saliva estimulada ^42^ (Tabla 1).

**Tabla 1:** Alteraciones relevantes causadas por la obesidad en las glándulas salivales. Muestra los principales cambios morfológicos y funcionales en el modelo animal y humano.

Referencias del estudio	Resultados	Glandulas salivares	Sujetos
Inoue *et al*. ^(^^24^^)^	Disminución del peso de las glandulas	-	Modelo experimental (ratas)
Mazaffari *et al*. ^(^^25^^)^	Cambios proinflamatorios	submandibular	Modelo experimental (ratas)
Renzi *et al*. ^(^^26^^)^	Disminuye la masa glandular, Hipertrofia de los acinos, increment de los ductos	submandibular	Modelo experimental (ratas)
Bozzato et al. ^(^^27^^)^	Incremento de los adipocitos	parotida	-
Pannuzio *et al*. ^(^^32^^)^	Alteración en la concentratión de fosfato, acido sialico, proteína y actividad de la peroxidasa	-	Humanos (niños)
Hordiienko *et al*. ^(^^33^^)^	Incremento en substancias reactivas al acido tiobarbiturico	-	Modelo experimental
Beregova *et al*. ^(^^16^^)^	Incremento en substancias reactivas al acido tiobarbiturico		Modelo experimental
Modéer *et al*. ^(^^36^^)^	Disminución total del estimulo del flujo salival	-	Humanos (niños)
Choromanska *et al*. ^(^^40^^)^	Disminución total del estimulo del flujo salival, disminución de la actividad de la peroxidasa,del fosfato y el acido sialico	-	Humanos
Guaré *et al*. ^(^^41^^)^	disminución de la actividad de la peroxidasa,del fosfato, el acido sialico y la concentración de la proteína e Ig	-	Humanos (niños y adolescentes)
de Campos *et al*. ^(^^42^^)^	Disminución del estimulo salival y la concentración de urea, fosfato y calcio	-	Humanos (niños)

## Obesidad y patologías prevalentes de la cavidad bucal.

Las glándulas salivales y la saliva desempeñan un papel importante en el mantenimiento de la salud bucal, ayudando a desarrollar y mantener la salud de los tejidos blandos y duros. Cuando se reduce el flujo de saliva, pueden desarrollarse problemas de salud bucal, como caries e infecciones orales.

### Obesidad y Caries Dental 

En dientes sanos, la pérdida de minerales se equilibra con los mecanismos de reparación de la saliva, por lo que si la saliva está presente en menor cantidad y calidad, podemos contribuir a un proceso de caries. Si bien, la relación entre caries y obesidad, no es clara, la literatura indica que la obesidad está ciertamente asociada con la aparición de caries tempranas en la infancia y la pubertad ^43^^-^^46^, aunque al ser dos patologías que coexisten en el tiempo y comparten factores etiológicos comunes, esto explicaría en parte la falta de asociación encontrada por algunos autores al estudiar diversas poblaciones ^47^^-^^52^. Modéer *et al.* ^(^^36^, reportaron que la obesidad de los niños/adolescentes se correlacionó significativamente con la cantidad de superficies dentales afectadas por la caries, así como los índices de placa y gingivitis. Los estudios realizados en adultos con sobrepeso grave, obesidad y diabetes coexistente indican una frecuencia significativamente más alta de caries en estos pacientes en comparación con el grupo control, de personas con diabetes sin obesidad ^53^^,^^54^. Datos similares a los reportados por Yao *et al.* ^(^^55^*,* quienes reportaron un efecto significativo de la obesidad sobre la prevalencia de caries en niños de educación primaria, en población China. Sin embargo, es difícil determinar claramente si esto se debe únicamente al sobrepeso o a desordenes en la dieta e higiene. Aunque según Prpić & Pezelj-Ribarić ^56^, el resultado de una dieta desequilibrada rica en carbohidratos que estimula el desarrollo de *Lactobacillus* sp. y *Streptococcus*, favorece el desarrollo de la caries en humanos. Por otra parte, estudios realizados por Costa *et al*. ^(^^57^, confirmaron la existencia de una correlación entre la obesidad y caries; el estudio realizado por estos investigadores en un grupo de niños (edad media de 6 años) de familias con bajos ingresos, reveló que más del 50% de los participantes tenían caries, y el 25% de estos niños eran obesos. Sin embargo, el nivel de ingreso socioeconómico familiar fue el factor más fuerte en la determinación de la existencia de caries. 

Por otra parte, Lehmann-Kalata *et al.*^58^, reportaron una mayor incidencia de caries dentales, peor estado de las encías e higiene oral en los pacientes obesos, en comparación con los de peso normal, además estos últimos tenían una cantidad significativamente mayor de saliva estimulada y en descanso que los pacientes con obesidad; existiendo una correlación estadísticamente significativa entre el aumento de los niveles de *Streptococcus mutans* y *Lactobacillus* spp. en pacientes obesos. Farsi *et al.*, señalaron valores distintos, estableciendo que para los dientes primarios y permanentes combinados, los niños con mayor índice de masa corporal y circunferencia de cintura presentaron una menor prevalencia de caries (*p* <0.05) ^59^. Por su parte Cereceda *et al.* ^(^^52^*,* no encontraron asociación entre caries y obesidad/sobrepeso, estos investigadores estudiaron un grupo de estudiantes de 5 a 15 años de escuelas públicas chilenas de nivel socio económico medio, no encontrando relación entre IMC y caries dental, ni entre sexos.

 En la revisión sistemática llevada a cabo por González *et al.* ^(^^45^*,* indicaron que la asociación entre caries y obesidad/sobrepeso, estaría explicada por el aumento de peso registrado debido a la dieta, sobre todo por la elevada frecuencia de consumo de azúcares y comidas entre horas, que da lugar a un incremento en el número de microorganismos cariogénicos. Por otra parte, la falta de asociación entre estas dos variables se debería a problemas en el tamaño muestral reducido, asociado a un corto periodo de seguimiento, además de valores infraestimados que no reportan lesiones o pérdida de dientes por caries. 

Por otro lado, la asociación inversa entre caries y obesidad/sobrepeso ^60^ estaría explicada por que a medida que progresan las caries y se hacen más dolorosas, limitarían la función masticatoria, disminuyendo así la ingesta de alimentos; así como factores biológicos, genéticos, socieconómicos, culturales, dietarios, y medioambientales ^49^^,^^61^.

La función salival se reduce en pacientes con obesidad y sobrepeso, lo que podría afectar el curso de la caries dental. Existen muchos estudios realizados en los que se establece una relación entre la obesidad y la caries dental; otros autores indican que al ser patologías que coexisten con el tiempo y comparten factores etiológicos, esta relación no se pudo establecer debido a la naturaleza multifactorial de la caries dental. La disminución en el flujo y la calidad de la saliva en pacientes obesos es clara, lo que sin duda causará algún efecto en el proceso de remineralización, un factor importante en la progresión de la caries dental. La necesidad de más estudios, tanto experimentales como prospectivos, podría ayudar a explicar esta asociación.

### Obesidad, Microbiota oral y periodonto

La microbiota humana sana está compuesta por gran cantidad de microorganismos ^62^, existiendo distintos factores que influyen en su composición, tales como la edad, la dieta, los antibióticos y la mayoría de los elementos de un estilo de vida moderno, además de ciertas enfermedades. Desde el nacimiento, el microbioma y el sistema inmune del huésped se codesarrollan y son mutuamente interdependientes ^(^^63^. Así, la microbiota moldea el desarrollo del sistema inmunológico y, a su vez, el sistema inmunológico configura la composición de la microbiota, como lo observado entre cambios sostenidos en la microbiota intestinal y su asociación con la obesidad ^64^ y la resistencia a la insulina ^62^. Existen otros estudios que describen una colonización microbiológica alterada en intestino de sujetos obesos, indicando que estos poseen más Firmicutes y relativamente menos Bacteroidetes en el intestino en comparación con los participantes con control de peso normal ^65^^,^^66^.

Hasta ahora no hay claridad en la relación entre la obesidad y la microbiota oral. Sin embargo, en estudios en modelo animal, se ha informado que la obesidad interfiere con la capacidad del sistema inmune para responder apropiadamente a la infección por el patógeno periodontal *Porphyromonas gingivalis*^67^. Además, se ha descrito una colonización mejorada de *Tannerella forsythia* en biofilm subgingival en sujetos obesos ^68^. Las adipocinas circulantes pueden influir en la respuesta inmune a nivel de la mucosa, tanto en la cavidad oral como en el intestino, afectando de este modo a la colonización microbiana. Por su parte, Ley *et al*. ^(^^63^, reportaron que los individuos obesos presentan diferencias en su microflora gatrointestinal en comparación a los normopesos infiriendo además que la flora cambia cuando se pierde peso. Otros autores ^68^^-^^70^, indicaron que la cavidad oral de los individuos obesos tiene niveles más altos de varias bacterias, cuando son comparados con controles no obesos, pareciendo probable que esas especies bacterianas podrían servir como indicadores biológicos de una condición de sobrepeso en desarrollo. Shillitoe *et al.* ^**(**^ ^**71**^*,* señalan que existe variación de las bacterias de la cavidad oral en pacientes obesos, posteriormente a la realización de cirugía bariátrica. Las alteraciones existentes en la microbiota de obesos puede estar relacionada con la concentración de adipocinas circulantes, las cuales pueden influir en la respuesta inmune a nivel de la mucosa tanto en la cavidad oral como en el intestino, afectando de este modo a la colonización microbiana ^**70**^. Con respecto a las bacterias periodontales Maciel *et al.*^**72**^, establecieron que los pacientes obesos con periodontitis crónica tuvieron niveles más altos y / o mayores proporciones de varios patógenos periodontales que los normopeso *Aggregatibacter actinomycetemcomitans*, *Eubacterium nodatum, Fusobacterium nucleatum ss vincentii, Parvimonas micra, Prevotella intermedia, Tannerella forthytia, Prevotella melaninogenica* y *Treponema socranskii*. Las proporciones de la mayoría de estos patógenos, así como *Campylobacter rectus* y *Eikenella corrodens*, se incrementaron en los sitios enfermos de los pacientes obesos en relación a los normopeso. Por otra parte Lehmann-Kalata *et al.* ^**(**^ ^**58**^*,* reportaron una correlación estadísticamente significativa entre el aumento de los niveles de *Streptococcus mutans* y *Lactobacillus* spp. en pacientes obesos al ser comparados con pacientes normopesos.

Con respecto a la asociación entre obesidad y enfermedad periodontal, existe suficiente evidencia para establecer una asociación positiva significativa entre ambas ^73^; Modéer *et al*. ^36^, indicaron que la obesidad infantil se asocia con aumento de la inflamación gingival cuando son comparados con normopesos (*p* <0.001). Posibles causas de cómo afecta la obesidad a los tejidos periodontales, estaría dado por la secreción de citoquinas proinflamatorias, desde el tejido adiposo ^74^^-^^76^, además, la expansión de este mismo durante el aumento de peso restringiría los vasos sanguíneos, causando la migración de macrófagos hacia el periodonto. La combinación de las situaciones mencionadas anteriormente puede inducir una inflamación crónica de bajo grado generalizada. Lo cual podría ser acompañado por hipertensión exacerbando la carga inflamatoria inducida por obesidad ^77^. Al usar como parámetro el Indice de Masa Corporal (IMC), Saito *et al*. ^78^, en un estudio realizado en Japón se demostró una correlación estadísticamente significativa entre el IMC y la profundidad de las bolsas periodontales en mujeres con obesidad, y demostró que el IMC por encima de 30 kg/m^2^ aumentó en más del cuatro veces el riesgo de periodontitis. Otros estudios japoneses que analizaron el estado periodontal mediante el índice CPITN (Índice Comunitario Periodontal de Necesidades de Tratamiento), evaluando las necesidades de tratamiento periodontal, demostraron una correlación positiva entre la exacerbación de los síntomas que indican una progresión significativa de la enfermedad periodontal y el aumento del peso corporal medido por IMC. Aunque en estudios experimentales de periodontitis inducida en animales, se vio que no hubo diferencias significativas en la pérdida de hueso proceso alveolar entre los animales obesos y normales ^79^. 

Por su parte Zuza *et al.* ^(^^80^*,* al estudiar niños de entre 5 a 10 años vieron que los niños obesos mostraron proporciones significativas de los códigos uno y tres del Índice periodontal comunitario CPI (44.2% y 7.4%, respectivamente), en comparación con sujetos de peso normal (*p* <0.05). El índice de placa visible (Visible Plaque Index VPI) fue similar entre ambos grupos (*p* >0.05). El sangrado al sondeo fue mayor en pacientes obesos que en niños con peso normal (*p* <0.05), lo que estaría indicando que los niños obesos parecen ser más susceptibles a la enfermedad periodontal. Resultados similares a los encontrados por Scorzetti *et al.*^81^, quienes encontraron una asociación entre la obesidad y los indicadores de riesgo periodontal en los niños, tales como depósitos de placa y sangrado al sondaje. Con respecto a la asociación entre periodontitis y obesidad en adolescentes, Cavalcanti *et al*. ^(^^82^, luego de analizar un total de 559 individuos, establecieron que el 18.6% tenían sobrepeso y el 98.4% tenían alguna forma de cambios periodontales como: sangrado (34.3%), cálculo (38.8%), saco poco profundo (22.9%) y saco profundo (2.3%), existiendo una asociación entre la presencia de cambios periodontales y la obesidad (*p* <0.05). 

La obesidad y su importante componente inflamatorio contribuyen, sin duda, a aumentar la gravedad y las secuelas de la enfermedad periodontal. Como se describió anteriormente, hay muchos estudios que describen la asociación positiva entre el sobrepeso / obesidad y la enfermedad periodontal, principalmente debido a la acción constante de los mediadores químicos secretados por el tejido graso en la cavidad oral, lo que crea un ambiente proinflamatorio general y contribuye A un aumento de la enfermedad periodontal. Esto se agrega a la disminución de la secreción salival, que desempeña un importante papel antibacteriano y antiinflamatorio a nivel local.

## Conclusion

Esta revisión describe los principales mecanismos subyacentes en torno a la influencia de la obesidad en la morfología y la función de las glándulas salivales, y cómo estas alteraciones afectan el funcionamiento de otros componentes de la cavidad oral, y están asociadas a patologías de alta prevalencia, como caries y enfermedad periodontal. Dado el aumento en la prevalencia de la población con sobrepeso / obesidad en todo el mundo, estos son aspectos importantes a considerar.

El fondo presentado debería inducir la cooperación entre médicos y dentistas para aumentar la conciencia sobre la salud y mejorar las condiciones de la cavidad oral en pacientes con obesidad y sobrepeso.

Se necesita más investigación en esta área, dado que existen pocos estudios morfológicos, ultraestructurales y funcionales con respecto a los cambios relacionados con la obesidad que ocurren en las estructuras de la cavidad oral, particularmente en las glándulas salivales. Estos estudios morfofuncionales junto con estudios que se centran en otros aspectos permitirían diseñar nuevas terapias farmacológicas que ayudarían a mitigar los efectos causados ​​por la obesidad en la cavidad oral.
